# Pan-Cancer Analysis Shows Enrichment of Macrophages, Overexpression of Checkpoint Molecules, Inhibitory Cytokines, and Immune Exhaustion Signatures in EMT-High Tumors

**DOI:** 10.3389/fonc.2021.793881

**Published:** 2022-01-12

**Authors:** Jayesh Kumar Tiwari, Shloka Negi, Manju Kashyap, Sheikh Nizamuddin, Amar Singh, Arun Khattri

**Affiliations:** ^1^ Department of Pharmaceutical Engineering and Technology, Indian Institute of Technology (Banaras Hindu University), Varanasi, India; ^2^ Facultad de Ingeniería y Tecnología, Universidad San Sebastián, Concepción, Chile; ^3^ Department of Urology, Medical Center-University of Freiburg, Freiburg, Germany; ^4^ German Cancer Consortium (DKTK) Partner Site Freiburg, German Cancer Research Center (DKFZ), Heidelberg, Germany; ^5^ Schulze Diabetes Institute, Department of Surgery, University of Minnesota, Minneapolis, MN, United States

**Keywords:** epithelial–mesenchymal transition (EMT), The Cancer Genome Atlas (TCGA), tumor immune microenvironment (TIME), immune checkpoints, cytokines

## Abstract

Epithelial–mesenchymal transition (EMT) is a highly dynamic process that occurs under normal circumstances; however, EMT is also known to play a central role in tumor progression and metastasis. Furthermore, role of tumor immune microenvironment (TIME) in shaping anticancer immunity and inducing the EMT is also well recognized. Understanding the key features of EMT is critical for the development of effective therapeutic interventions. Given the central role of EMT in immune escape and cancer progression and treatment, we have carried out a pan-cancer TIME analysis of The Cancer Genome Atlas (TCGA) dataset in context to EMT. We have analyzed infiltration of various immune cells, expression of multiple checkpoint molecules and cytokines, and inflammatory and immune exhaustion gene signatures in 22 cancer types from TCGA dataset. A total of 16 cancer types showed a significantly increased (p < 0.001) infiltration of macrophages in EMT-high tumors (mesenchymal samples). Furthermore, out of the 17 checkpoint molecules we analyzed, 11 showed a significant overexpression (p < 0.001) in EMT-high samples of at least 10 cancer types. Analysis of cytokines showed significant enrichment of immunosuppressive cytokines—*TGFB1* and *IL10*—in the EMT-high group of almost all cancer types. Analysis of various gene signatures showed enrichment of inflammation, exhausted CD8+ T cells, and activated stroma signatures in EMT-high tumors. In summary, our pan-cancer EMT analysis of TCGA dataset shows that the TIME of EMT-high tumors is highly immunosuppressive compared to the EMT-low (epithelial) tumors. The distinctive features of EMT-high tumors are as follows: (i) the enrichment of tumor-associated macrophages, (ii) overexpression of immune checkpoint molecules, (iii) upregulation of immune inhibitory cytokines *TGFB1* and *IL10*, and (iv) enrichment of inflammatory and exhausted CD8+ T-cell signatures. Our study shows that TIMEs of different EMT groups differ significantly, and this would pave the way for future studies analyzing and targeting the TIME regulators for anticancer immunotherapy.

## Introduction

Epithelial cells are specialized cells that line the outer and inner surfaces of various organs throughout the body. They maintain the apico–basal axis of polarity and remain in close contact with each other. Conversely, mesenchymal cells are unspecialized cells capable of differentiating into any cell type in the body at any time. They do not have a polarity and are hence loosely organized in the extracellular matrix (ECM) (1). Under appropriate conditions, these two types of cells can transform into each other through complex biological processes, namely, epithelial–mesenchymal transition (EMT) and mesenchymal–epithelial transition (MET). EMT is a highly dynamic process that occurs during embryogenesis, tissue regeneration, and wound healing under normal circumstances. However, several studies have identified the role of EMT in causing tumor progression and metastasis (2). Tumor cells undergoing EMT have increased motility and invasiveness, which help them disseminate to distant sites and metastasize. In addition, they become resistant to apoptosis and anticancer drugs, contribute to immunosuppression, and act as cancer stem-like cells (1, 3).

EMT is not always complete in tumor cells, i.e., these cells can be in multiple transitional states and express mixed epithelial and mesenchymal markers (4). Such hybrid cells with incomplete EMT can be more aggressive than those with complete EMT and move in clusters (5–7). These transitional cells are considered to be primary mediators of therapy resistance, making the EMT process a promising target for therapeutic intervention to prevent these effects (8). Tumor immune microenvironment (TIME) acts as a significant stimulant in inducing the EMT in a tumor. The five main classes of stimuli that induce the EMT are as follows: hypoxia and low pH, innate and adaptive immune responses, mechanical stress, altered ECM, and treatment with antitumor drugs (1). Furthermore, the EMT-inducing microenvironment can also cause epigenetic changes and heritable effects that maintain the mesenchymal state even when the EMT-initiating microenvironment is no longer present (1).

The development of novel immune checkpoint inhibitors (ICIs) has provided patients of several different cancer types with treatment options that demonstrate better overall survival (OS) and progression-free survival (PFS) as compared to the standard of care therapies. In melanoma, tumors innately resistant to anti-Programmed cell death 1 (PD-1) therapy (IPRES or innate anti-PD-1 resistance) have an upregulation of the signature genes involved in EMT, cell adhesion, ECM remodeling, angiogenesis, and wound healing. Interestingly, treatment-resistant tumors show downregulation of epithelial marker gene *CDH1*, upregulation of mesenchymal transition genes, and upregulation of T cell-suppressive cytokine interleukin (IL)10 (9).

EMT plays a central role in shaping the TIME and immune escape, and its role has been analyzed in several cancer types individually, including melanoma and lung cancer (9, 10). However, the role of EMT has not been explored comprehensively. Given the central role of EMT in cancer progression and treatment, we have carried out a pan-cancer TIME analysis of TCGA dataset—including infiltration of various immune cells, expression of multiple checkpoint molecules and cytokines, and gene signatures—in context to EMT.

## Materials and Methods

### Study Cohort and Calculation of Epithelial–Mesenchymal Transition Scores

**Figure 1:**
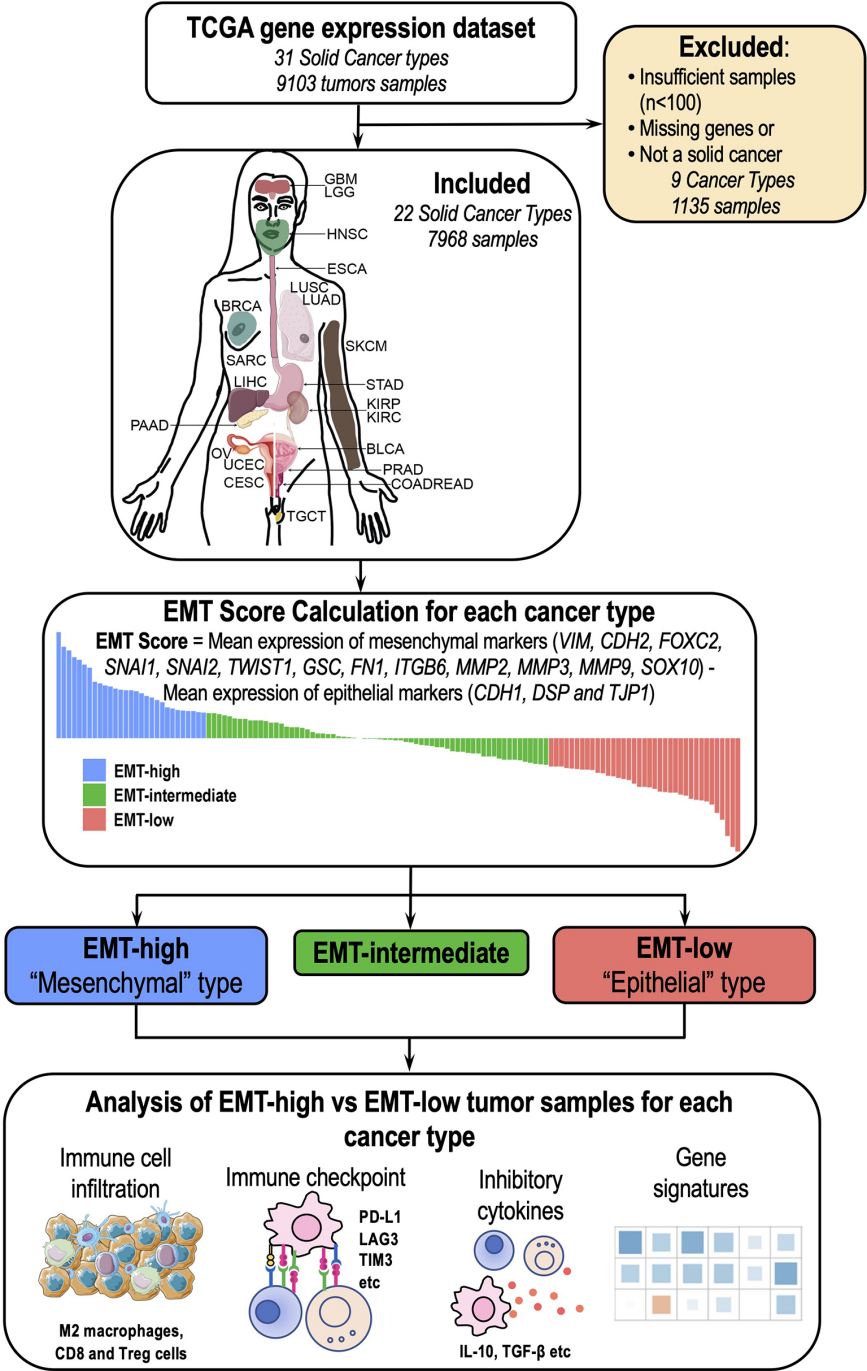
Overview of the study: A total of 22 cancer types (7,968 samples) were included in the study. Epithelial–mesenchymal transition (EMT) score was calculated for each of these 22 cancer types, and different aspects of tumor immune microenvironment (TIME) were analyzed between EMT-high and EMT-low samples.

Scaled (z-scores) gene expression data of The Cancer Genome Atlas (TCGA) samples was downloaded from the Broad Institute's Genome Data Analysis Center (GDAC) (http://gdac.broadinstitute.org/) for 23 cancer types. Solid tumors having sufficient samples (n >100) were included in this study. For EMT score calculation, we included 16 canonical markers as described elsewhere (10). EMT score was calculated by subtracting the mean z-scores of “Epithelial markers” (*CDH1*, *DSP*, and *TJP1*) from the mean z-scores of “Mesenchymal markers” (*VIM*, *CDH2*, *FOXC2*, *SNAI1*, *SNAI2*, *TWIST1*, *GSC*, *FN1*, *ITGB6*, *MMP2*, *MMP3*, *MMP9*, *SOX10*). The code used for EMT score calculation is available at the following link: https://github.com/snizam001/EpiMesen-ImmuneFilteration. Thyroid Carcinoma (THCA) cancer type was excluded because it did not have gene expression data of the *CDH1* gene, which was required for EMT score calculation. Therefore, we included a total of 22 solid tumor types from TCGA data (**Figure 1** and **Supplementary Table S1**). Tumor samples for each cancer type were categorized into three groups based on their EMT scores: the top 25 percentile samples having the highest EMT scores were classified as EMT-high (“mesenchymal” phenotype), the middle 26–75 percentile samples with medium EMT scores were classified as EMT-intermediate, and the lowest 75–100 percentile samples having the least EMT scores were classified as EMT-low (“epithelial” phenotype). Samples of all the three EMT groups were analyzed for the infiltration of various immune cells, expression of multiple checkpoint molecules and cytokines, and inflammatory and immune exhaustion gene signatures.

### Analysis of Immune Cell Infiltration, Expression of Immune Checkpoint Molecules, Inhibitory Cytokines, and Gene Signatures

The TIMEs of the samples categorized as EMT-high and EMT-low were analyzed for the infiltration of various immune cells. We chose 17 distinct immune cell types of both innate and adaptive immune systems to compare their infiltration in different cancer types (**Supplementary Table S2**). Precalculated enrichment scores for these immune cells were downloaded from the xCell website (https://xcell.ucsf.edu/) (11). The xCell enrichment scores for each immune cell were compared across the three EMT groups.

TIME of EMT-high and EMT-low samples was further analyzed for the gene expression of various immune checkpoints and inhibitory cytokines. Gene expression levels were compared between “mesenchymal” and “epithelial” groups for 17 immune checkpoint molecules (*PD1*, *PD-L1*, *PD-L2*, *CTLA4*, *LAG3*, *KIR*, *TIM3*, *VISTA*, *NOX2*, *SIGLEC5*, *SIGLEC7*, *SIGLEC15*, *FASLG*, *ICOS*, *GITR*, *TNFRSF4*, and *TNFRSF9*) (**Supplementary Table S3**) and 17 cytokine molecules (*IFNA1*, *IFNB1*, *IFNG*, *TNFA*, *TGFB1*, *IL1A*, *IL1B*, *IL2*, *IL3*, *IL4*, *IL5*, *IL6*, *CXCL8*, *IL10*, *IL12A*, *IL12B*, and *STAT6*) in all the 22 cancer types (**Supplementary Table S4**). We also analyzed various gene signatures (**Supplementary Table S5**) in all the three EMT group samples. Violin plots were prepared using the *ggplot2* package in R. Kolmogorov–Smirnov (KS) test (from *dgof* package in R) compared EMT-high and EMT-low samples, whereas one-way ANOVA was used to compare the three EMT groups.

### Principal Component and Survival Analysis

Principal component analysis (PCA) was performed on the median values of each TIME marker (immune cell enrichment, expression of checkpoint molecules and cytokines, and gene expression signatures) for each EMT group in each cancer type (**Supplementary Table S6**) using the *prcomp* function in R. The contribution of each TIME marker in PCA was calculated by R package *factoextra*, and conditional probabilities of high expression of the top 4 markers in PCA were calculated, given it belongs to a particular EMT group. Another PCA was carried out using the differences in the median values of each TIME marker between EMT-high and -low groups for each cancer type (**Supplementary Table S6**). Furthermore, the K-means clustering algorithm (R package *factoextra*) was used to ascertain the optimum number of clusters.

The OS and PFS data were downloaded from TCGA. We used the *survival* and *survminer* packages in R to plot the Kaplan–Meier survival curves. Statistical significance for OS and PFS was determined using the log-rank test.

## Results

### Distribution of Epithelial–Mesenchymal Transition Scores in Various Cancer Types

**Figure 2:**
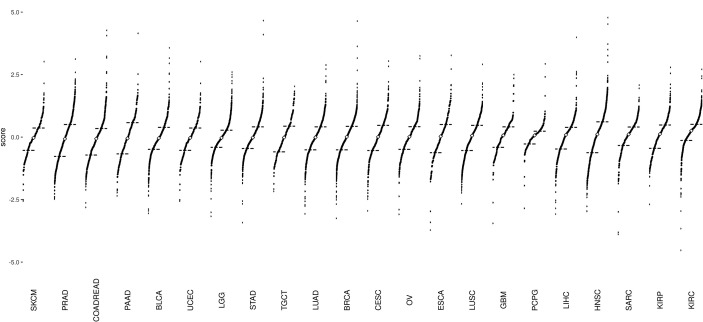
Divergent dot plots for the distribution of epithelial–mesenchymal transition (EMT) scores across 22 cancer types: The plots are arranged in increasing order of median values. The black dashes represent the end of the first quantile (25% mark) and the beginning of the fourth quantile (75% mark), respectively. The white diamond point corresponds to the median of EMT scores of all samples per cancer type. Skin Cutaneous Melanoma (SKCM) tumor samples have the lowest median EMT score, whereas Kidney Renal Clear Cell Carcinoma (KIRC) tumor samples have the highest median EMT score.

In the current study, we analyzed 7,968 tumor samples representing 22 distinct cancer types from TCGA dataset (**Figure 1** and **Supplementary Table S1**). Among all cancer types, Skin Cutaneous Melanoma (SKCM) showed the lowest median value, whereas Kidney Renal Clear Cell Carcinoma (KIRC) showed the highest median value (**Figure 2**). The EMT score in Prostate Adenocarcinoma (PRAD) had maximum dispersion [interquartile range (IQR) = 1.275], whereas Pheochromocytoma and Paraganglioma (PCPG) had the least dispersion (IQR = 0.51).

### Monocytes, Macrophages, and Some Other Immune Cells Were Enriched in Epithelial–Mesenchymal Transition-High Tumors

**Figure 3:**
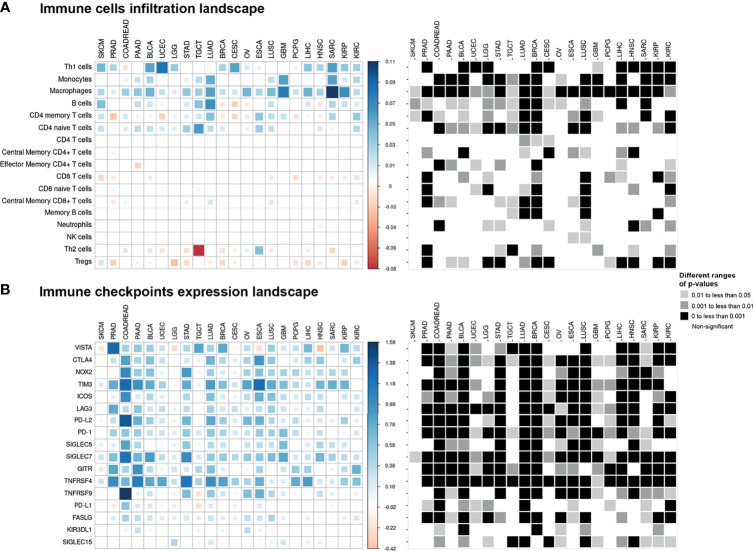
Pan-cancer immune cell infiltration and immune checkpoint expression landscape: The colored squares represent the difference between: **(A)** the medians of immune cell enrichment score of epithelial–mesenchymal transition (EMT)-high (mesenchymal) and EMT-low (epithelial) samples of each cancer type and **(B)** the medians of expression of immune checkpoints in EMT-high and EMT-low samples of each cancer type. The larger sized and darker blue or red shaded squares correspond to a greater difference. Grayscale squares on the right side represent the corresponding p-values for differences between EMT-high and EMT-low groups of tumors.

**Figure 4:**
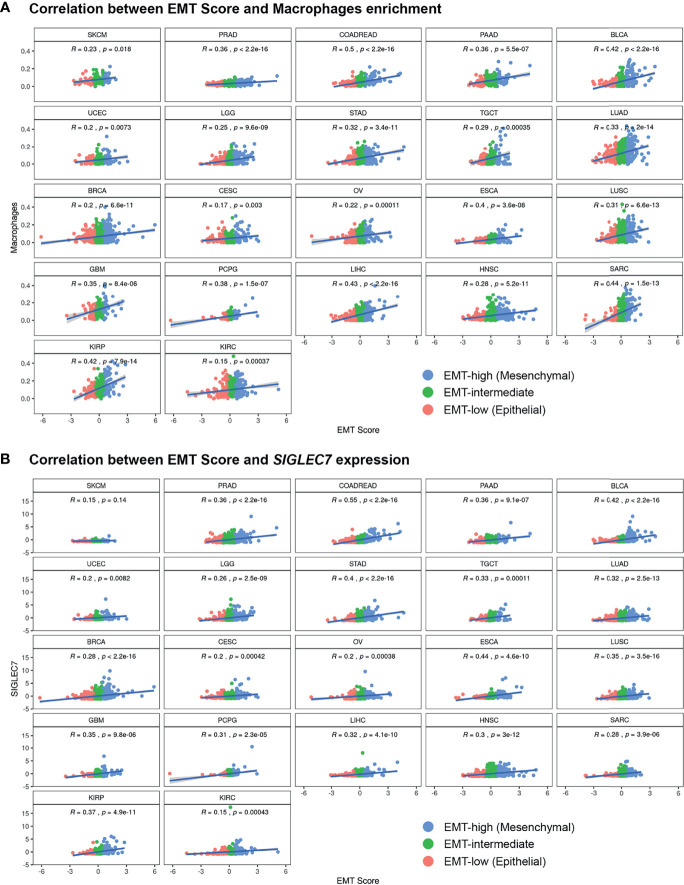
Pan-cancer correlation analysis of EMT Score with **(A)** xCell enrichment score of Macrophages and **(B)** gene expression values of immune checkpoint gene SIGLEC7, in all three EMT groups.

**Figure 5:**
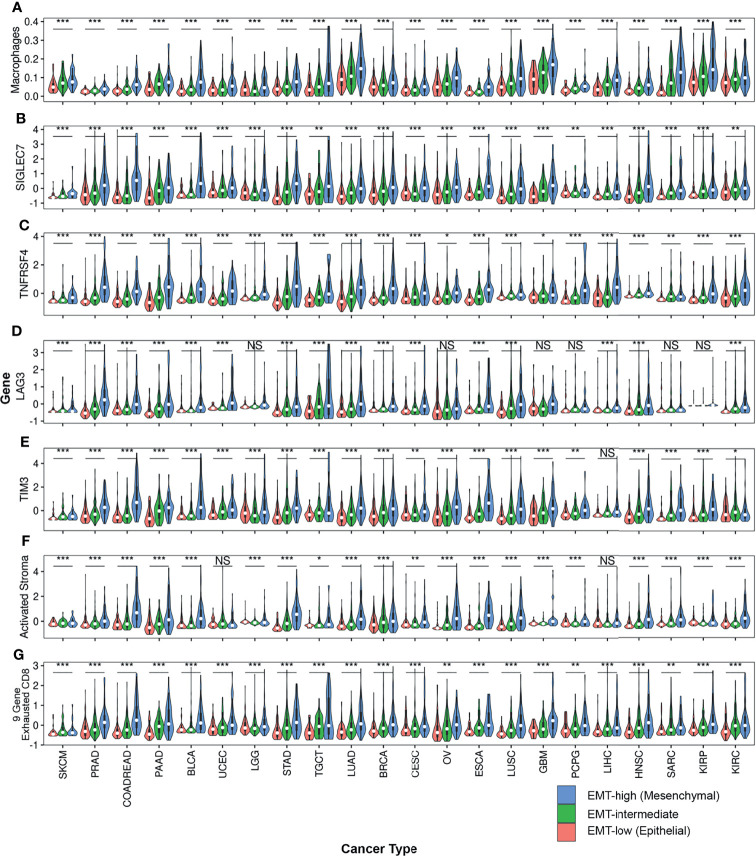
Violin plots comparing expression of various TIME markers among all three EMT groups across all cancer types: Violin plots of **(A)** Macrophages, **(B)** SIGLEC7, **(C)** TNFRSF4, **(D)** LAG3, **(E)** TIM3, **(F)** Activated Stroma and **(G)** 9-Gene Exhausted CD8+ T-cells are represented. One-way anova was used to compare the expression of these TIME markers. Asterisks (*) indicate significance based on p-values. *** represents p-value < 0.001, ** represents p-value between 0.001 to <0.01, * represents p-value between 0.01 <0.05, and finally NS represents non-significant p-value.

Comparison of enrichment scores of immune cell infiltration between EMT-low and EMT-high samples showed that several tumor types with high EMT score (mesenchymal phenotype) had a significantly increased infiltration of monocytes and macrophages (**Figure 3A**). A total of 16 cancer types showed a significantly increased (p < 0.001) enrichment of macrophages in EMT-high samples compared to EMT-low. Enrichment of macrophages showed a significantly positive correlation with EMT score for all the cancer types (**Figure 4A**), and when all three EMT groups were compared, all the cancer types showed significant differences (**Figure 5A**). Several tumor types showed significantly higher enrichment of Type 1 helper T cells (Th1) in EMT-high tumors. On the other hand, Type 2 helper T cells (Th2) showed significantly higher enrichment (p < 0.001) in Testicular Germ Cell Tumor (TGCT) and mild enrichment in EMT-low samples of some other cancer types except for Esophageal Carcinoma (ESCA). As opposed to EMT-high tumors, only a few immune cell types showed enrichment in EMT-low tumors: regulatory T cells (Tregs) were moderately enriched in several tumor types with low EMT scores (p < 0.001 in 9 tumor types) (**Figure 3A**). Similarly, CD8+ T cells were mildly enriched in EMT-low samples of some cancer types.

### Most Checkpoint Molecules, Including *SIGLEC7, TNFRSF4, LAG3*, and *TIM3*, Showed Significantly Higher Expression in Epithelial–Mesenchymal Transition-High Tumors

Except for SKCM, we observed a consistent trend of positive correlation between EMT score and expression of checkpoint molecules regardless of cancer type (**Figure 3B**). Of the 17 checkpoint molecules we analyzed, 11 showed a significant overexpression (p < 0.001) in EMT-high samples of at least 10 cancer types compared to EMT-low samples. Interestingly, mesenchymal tumor samples of all cancers, except TGCTs, showed a significantly higher (p < 0.05) expression of *SIGLEC7* (**Figure 3B**). Similarly, the expression of *TNFRSF4* was significantly higher (p < 0.001) in EMT-high samples of all cancer types, except for SKCM and Glioblastoma. Even *LAG3* showed a significantly higher expression (p < 0.05) in EMT-high tumors of all cancers, except SKCM, TGCT, and Sarcoma (SARC). Furthermore, it was noticed that SKCM mesenchymal tumors showed little or no significant enrichment of any checkpoint marker, whereas Bladder Urothelial Carcinoma (BLCA) and Colorectal Adenocarcinoma (COADREAD) were enriched in all checkpoint markers. Correlation analysis of EMT score with *SIGLEC7* showed a significantly positive correlation for all cancer types except SKCM (**Figure 4B**), and *TIM3* expression showed a significantly positive correlation for most cancer types (**Supplementary Figure S1**). Comparing these checkpoint genes among the three EMT groups showed a highly significant difference for almost all cancer types, with EMT-high samples having the highest expression (**Figures 5B–E**).

Though many cancer types showed a consistent trend of increased expression of most checkpoints with an increase in EMT score, mesenchymal Lung Adenocarcinoma (LUAD) tissues were significantly enriched in all immune checkpoint markers (except *KIR3DL1*) (**Figure 3B**). Similarly, in mesenchymal Lung Squamous Cell Carcinoma (LUSC) tumors, all except *GITR* (*TNFRSF18*) and *PD-L1* (*CD274*) genes were significantly high. Surprisingly, in more than half of the cancer types, *PD-L1* expression did not show any association with EMT score.

### Immunosuppressive Cytokines *TGFB1* and *IL10* Showed Significantly Higher Expression in Epithelial–Mesenchymal Transition-High Tumors

**Figure 6:**
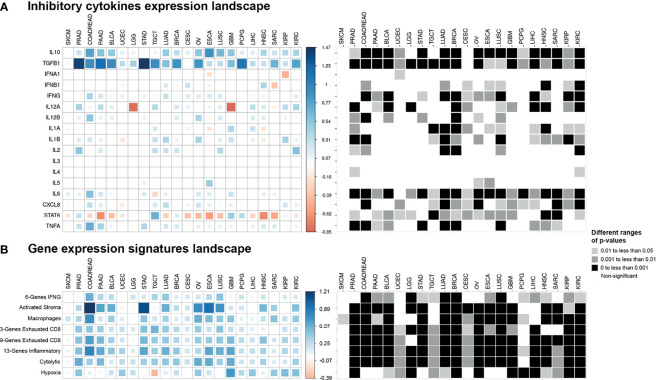
Pan-cancer cytokines expression and gene expression signatures landscape. The colored squares represent the difference between: **(A)** the medians of expression of cytokines in EMT-high and EMT-low samples of each cancer type and **(B)** the medians of gene expression signatures in EMT-high and EMT-low samples of each cancer type. The larger sized and darker blue or red shaded squares correspond to the greater difference. Greyscale squares on the right side represent the corresponding p-values for differences between EMT-high and EMT-low groups of tumors.

Immunosuppressive cytokine markers *TGFB1* and *IL10* were significantly enriched in the EMT-high group of almost all cancer types compared to those in the EMT-low (**Figure 6A**). These genes showed a highly significant positive correlation with EMT score, and this difference remained significant when all three EMT groups were considered for comparison (**Supplementary Figures S2E, F**); In contrast, for *IL3*, *IL4*, *IL5*, and *IFNA1*, there was no discernible pattern of altered expression in either mesenchymal or epithelial type tumors (**Figure 6A**). Similar to our observation in checkpoint molecules, SKCM tumor samples showed no significant expression for any cytokine to be associated with EMT score.

In addition to *TGFB1* and *IL10*, gene expression of *IFNG*, *IL6*, and *CXCL8* were all significantly increased (p < 0.05) in mesenchymal samples for more than half of the cancer types (**Figure 6A**). We also observed a strong association of *IL12A* expression with low EMT score in Brain Lower-Grade Glioma (LGG, p < 0.001) and Glioblastoma Multiforme (GBM, p < 0.01) cancer types. Moreover, only *STAT6* showed significantly higher expression (p < 0.05) in epithelial samples of most cancer types.

### Epithelial–Mesenchymal Transition-High Tumors Are Enriched in Inflammation, Exhausted CD8+ T Cells, and Activated Stroma Signatures

Analysis of gene signatures showed a trend toward increased inflammation and exhaustion in EMT-high tumors. EMT-high tumors showed enrichment of 6-gene interferon gamma (IFNG) and 13-gene inflammatory signatures (**Figure 6B**). As compared to EMT-low, EMT-high samples of at least 16 cancer types showed significantly increased enrichment of activated stromal signature, especially COADREAD, Stomach Adenocarcinoma (STAD), Ovarian Serous Cystadenocarcinoma (OV), ESCA, and LUSC. Exhausted CD8+ T cells were also enriched in EMT-high tumors, with at least 16 cancer types showing a highly significant enrichment (p < 0.001) of 9-gene exhausted CD8+ T cells (**Figure 5G**) and 13 cancer types showing a highly significant enrichment of 3-gene exhausted CD8+ T cells (**Figure 6B**). Additionally, 16 EMT-high tumors showed enrichment of Cytolytic activity signature (p < 0.001). Most tumor types showed enrichment of Hypoxia signature in EMT-high samples except for TGCT, which showed increased Hypoxia signature in EMT-low tumors (p < 0.01). Gene signature analysis also confirmed the enrichment of macrophages in EMT-high tumors, as observed in the enrichment analysis by xCell (**Figures 3A**, **6B**). Most of the gene signatures showed a significantly positive correlation with EMT score (**Supplementary Figure S3**), and when all three EMT groups were compared, a significant difference was observed for almost all cancer types (**Figures 5F, G**).

### Clustering Different Cancer Types by Tumor Immune Microenvironment

**Figure 7:**
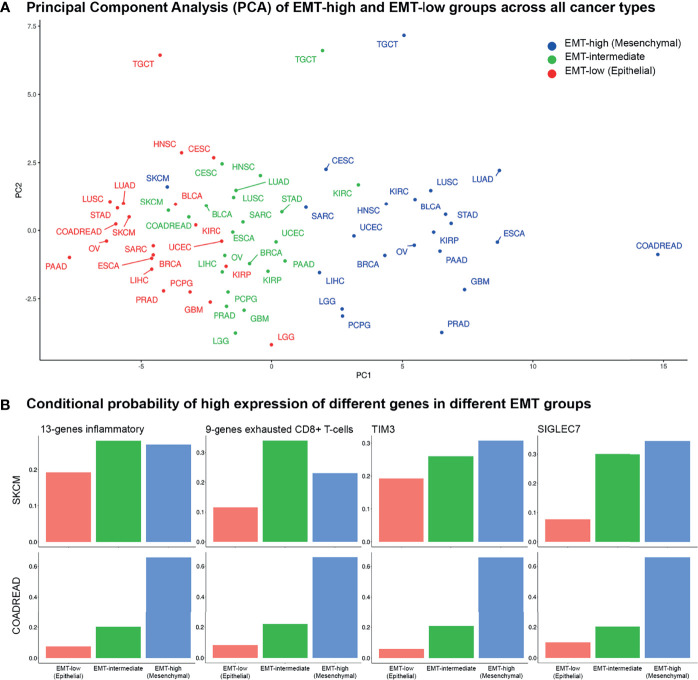
Principal component and conditional probability analysis: **(A)** Principal component analysis (PCA) of tumor immune microenvironment (TIME) markers across all cancer types in all three epithelial–mesenchymal transition (EMT) groups. Skin Cutaneous Melanoma (SKCM) patients belonging to the three EMT groups cluster together, whereas for COADREAD, they are far apart. **(B)** Conditional probabilities of high expression of top 4 TIME markers in PCA given it belongs to a given EMT group.

PCA using median expression values of each TIME marker showed coclustering of EMT-high, -intermediate, and -low samples of most cancer types (**Figure 7**). Interestingly, samples of the three EMT groups of SKCM cluster together, while for COADREAD, they were far apart, recapitulating the results of individual TIME markers. The 13-gene inflammatory and 9-gene exhausted CD8+ T-cell signatures, *TIM3*, and *SIGLEC7* were the top contributing factors in PCA (**Supplementary Table S7**). The conditional probability of high expression of 13-gene inflammatory signature or *TIM3*, given it belongs to a particular EMT group, showed a similar probability for the three EMT groups of SKCM. In contrast, for COADREAD, the probability matched with the EMT group, i.e., it was low, intermediate, and high for low, intermediate, and high EMT groups, respectively (**Figure 7B**). For the 9-gene exhausted CD8+ T cells, the EMT-intermediate samples of SKCM had the highest probability, while for COADREAD, the probability matched with the EMT group. For *SIGLEC7*, the probabilities in EMT-high and EMT-intermediate groups of SKCM were similar, while for COADREAD, it matched with the EMT group (**Figure 7B**).

**Figure 8:**
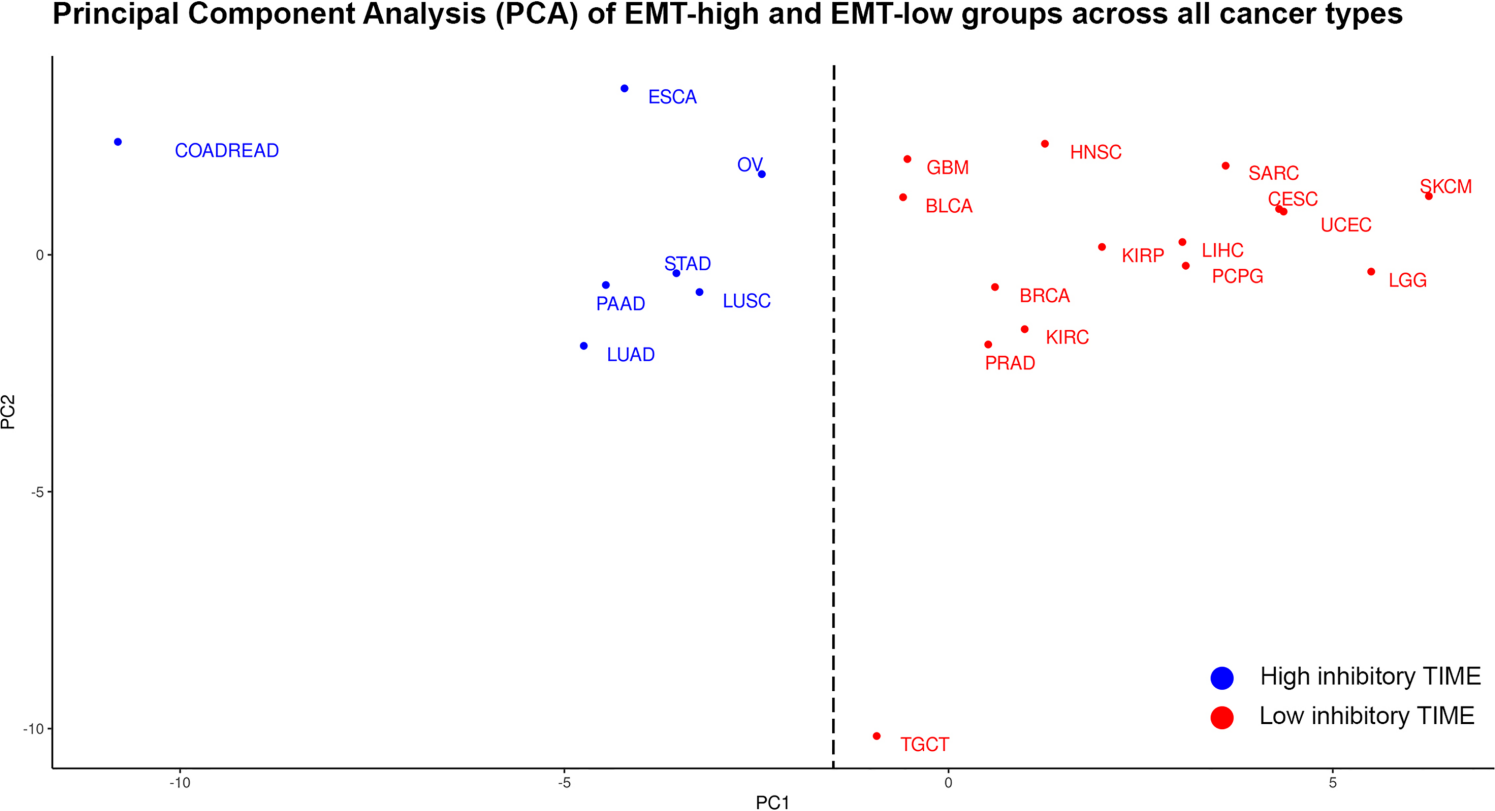
Principal component analysis (PCA) of differences in the median values of each tumor immune microenvironment (TIME) marker between epithelial–mesenchymal transition (EMT)-high and EMT-low groups of each cancer type: Cancer types in blue are classified as having high inhibitory TIME in EMT-high tumors as compared to EMT-low, while cancer types in red are classified as having not-highly inhibitory TIME in EMT-high tumors as compared to EMT-low.

To classify cancers based on the inhibitory TIME, the differences in the median values of each TIME marker between EMT-high and -low groups were used. The Silhouette method showed that the cancers could be optimally classified into two (K = 2) groups (**Supplementary Figure S4**). Based on the Principal Component 1 (PC1) in PCA and K-means clustering, the clusters were confirmed: (a) cancer types in which TIME was highly inhibitory in EMT-high tumors as compared to EMT-low and (b) cancer types in which TIME was not highly inhibitory in EMT-high tumors as compared to EMT-low samples of the same cancer type (**Figure 8**).

### Survival of Patients of Different Epithelial–Mesenchymal Transition Groups

Comparison of patient survival from EMT-high and EMT-low groups of different cancer types showed a significantly better OS of EMT-low (epithelial) patients of LGG, Head and Neck Squamous Cell Carcinoma (HNSC), and KIRC (**Supplementary Figure S5A**). Similarly, analysis of progression-free interval (PFI) showed significantly better PFI of EMT-low patients of PRAD, LGG, HNSC, Kidney Renal Papillary Cell Carcinoma (KIRP), and KIRC (**Supplementary Figure S5B**). Interestingly, all these cancers grouped to a Low inhibitory TIME group in PCA analysis (**Figure 8**).

When patients of all three EMT groups were considered, a significant difference in OS was observed for LGG, OV, LIHC, and KIRC (**Supplementary Figure S6A**). Analysis of PFI showed significant differences for PRAD, BLCA, LGG, HNSC, SARC, and KIRC (**Supplementary Figure S6B**). Of these observations, the differences were highly significant for LGG and KIRC. Interestingly, OS and PFI of EMT-intermediate LGG patients were significantly better than those of the other two groups, while EMT-intermediate patients of KIRC had moderate survival.

## Discussion

We have comprehensively analyzed the differences in TIME of patients of three EMT groups of 22 cancer types in TCGA data. Apart from its role in embryonic development and tissue fibrosis, EMT plays a pivotal role in tumor immunosuppression and immune evasion (12). Our analysis of infiltrating immune cells in TIME showed significantly increased enrichment of Th1 cells, monocytes, and macrophages in EMT-high samples of several tumor types. Among these cell types, enrichment of macrophages was consistent across all tumor types. The gene signature analysis confirmed the enrichment of tumor-associated macrophages (TAMs) in EMT-high tumors compared to other EMT groups. TAMs are considered key cells that promote inhibitory TIME by producing inhibitory cytokines, chemokines, and growth factors and trigger inhibitory immune checkpoint protein release in T cells (13). Recent studies have suggested that TAMs involved in the regulation of EMT and macrophages recruited at the tumor site promote tumor growth by enhancing EMT progression (13–15). Infiltration of CD8+ T cells was mildly reduced from the TIME of EMT-high tumors of several cancer types, which is in line with the previous study on lung cancer (10). This study also showed decreased infiltration of CD4+ T cells in mesenchymal lung cancer (10); however, we did not see decreased CD4+ T-cell infiltration in the EMT-high tumors of most cancer types we analyzed.

The T helper (Th) CD4+ T cells and the cytotoxic (Tc) CD8+ T cells are the key players that mediate the adaptive anticancer immune response and, along with TAMs, are the most abundant cells present in the TIME of several cancer types (16, 17). Upon antigen encounter, both CD4+ and CD8+ T cells differentiate into committed subgroups of cells. The helper cells differentiate into various lineages—Th1, Th2, and Th17—depending upon the type of cytokines released at the site of activation. Similarly, CD8+ T cells can also differentiate into T cytotoxic cell type 1 (Tc1) and T cytotoxic cell type 2 (Tc2) depending upon type 1 or type 2 cytokine response (16, 18). Transforming growth factor (TGF)β and IL6—responsible for the above differentiation processes—were found to be significantly high in several EMT-high tumors. After encountering cancer antigens, T cells get activated and migrate to the TIME and CD8+ T cells evolve into cytotoxic T lymphocytes (CTLs) and exert their antitumoral activity, resulting in the destruction of tumor cells (19). The antitumoral response is supported by CD4+ Th1 cells *via* secretion of pro-inflammatory cytokines [i.e., interferon (IFN)γ, tumor necrosis factor (TNF)α and IL2], inducing T-cell activation and CTL cytotoxicity, as well as the antitumoral activity of natural killer (NK) cells and macrophages (20). In our analysis, several tumor types showed significantly higher enrichment of Th1 cells in EMT-high tumors. On the other hand, Th2 cells showed significantly higher enrichment in TGCT and mild enrichment in EMT-low samples of some other cancer types (**Figure 3A**). Th1 cells are known to produce IFNγ and TGFβ which activate macrophages (21). Our data indicate that EMT-high tumors lack CTL cytotoxic activity and hence tumor clearance due to limited support of IFNγ, TNFα, and IL2 and abundance of suppressive IL10 and TGFβ cytokines.

As tumors progress, recruitment of Tregs takes place in TIME. Tregs suppress the development, activation, and cytotoxicity of effector immune cells, such as Th1, CTLs, macrophages, and NK cells (22). Tregs regulate immune response by several mechanisms including immunoregulatory cytokines IL10 and TGFβ and cytolysis of effector cells by production of perforin and granzyme (23). We noticed significantly higher expression of Tregs in EMT-low tumors of nine tumor types (**Figure 3A**) as compared to EMT-high tumors, whereas TAMs and Th1 cells were significantly higher in several EMT-high tumors. These observations indicate that the TIMEs of EMT-high and EMT-low tumor display distinct subsets of suppressive immune cells.

Previous studies on non-small cell lung cancer and Pancreatic Adenocarcinoma (PAAD) have shown the association of Forkhead box P3 (FOXP3) expression (marker of Tregs) with poor survival (24, 25). In our analysis, we noted mild enrichment of Tregs in EMT-low patients of PAAD and LUAD, but LUSC did not show any difference. Survival analysis of different EMT groups of these cancer types did not show a significant difference (**Supplementary Figures S5, S6**). These findings should be experimentally verified, as our immune cell enrichment results are based on the xCell algorithm, which utilizes gene expression values to predict enrichment of various immune cells. Nevertheless, the survival analysis of some other cancer types especially KIRC and LGG showed highly significant difference in both OS and PFI; in both of these cancer types, EMT-high patients showed worse survival (**Supplementary Figures S5, S6**), which is in line with previous studies (26–28).

There was a general trend toward higher expression of checkpoint molecules in EMT-high tumors, and several checkpoint molecules showed significantly higher expression in EMT-high tumors. In our analysis, immune inhibitory checkpoint genes, *LAG3*, *SIGLEC7*, *PD-1*, *PD-L2*, *TIM3*, and others, showed significantly higher expression in EMT-high samples of several cancer types. A previous report on LUAD has shown higher expression of immune inhibitory checkpoint molecules *PD-L1*, *PD-L2*, *PD-1*, *TIM-3*, *B7-H3 (CD276)*, *BTLA*, and *CTLA-4* in EMT-high tumors (29). Our findings corroborate these results in several other cancer types, even though the genes used for the EMT signature in both of these studies are different (29). Another report on LUAD has shown similar findings (10). In addition, we have noted the overexpression of immune stimulatory checkpoint genes *ICOS*, *TNFRSF4*, and *TNFRSF9* in EMT-high samples. These findings are also in agreement with the earlier study (10).

Analysis of cytokines showed that the *IL10* and *TGFB1* (TGFβ) were overexpressed in EMT-high tumors of most cancer types, and both of these are known to suppress antitumor immunity in TIME. TAMs, anti-inflammatory macrophages, and Tregs are the key source of highly suppressive cytokines IL10 and TGFβ (30). Both of these cytokines induce Tregs and inhibit dendritic cell function to present tumor antigens to activate CD4+ and CD8+ T cells (31, 32). TGFβ is the central inflammatory cytokine in TIME, and its role in mediating EMT in different types of cancer has been extensively studied and well-established (33–35). Moreover, it has been shown that both *TGFB1* and *IL-10* together, but not alone, can suppress B-cell activation induced by toll-like receptor (TLR) stimulation (36). The production of inflammatory cytokines in TME plays an active role in supporting inflammation and meditating tumor progression and EMT (37). Previous *in vitro* study on epithelial cancer cells has shown that cancer cell lines of different origins—when incubated with either supernatant derived from a mixed lymphocyte population or a mix of inflammatory cytokines (TNFα, TGFβ and IFNγ)—undergo a series of changes typical of the EMT. These cell lines show notable enhancement of *snail1* and *snail2* gene transcription and downregulation of *CDH1* (E-cadherin) expression, accompanied by an upregulation of *VIM* (Vimentin) (38). This and other studies have demonstrated that various inflammatory cytokines—including TNFα IL6, CXCL8 (IL8), and most importantly TGFβ—are the main determinants of EMT induction (37, 39). In our pan-cancer analysis, we found a strong and significant correlation between inflammatory (IL6, CXCL8, IL12, TGFβ) and suppressive cytokines (IL10) with EMT score (**Figure 6A** and **Supplementary Figures S1B, S2F**). Altogether, our analysis further substantiates the key role of inflammatory cytokines in EMT and align well with *in vitro* and experimental findings of previous studies.

Analysis of gene signatures showed EMT-high tumors have increased inflammation and exhaustion. Although immune cell infiltration analysis showed mildly reduced CD8+ T cells in EMT-high tumors (**Figure 3A**), exhausted CD8+ T cell signature was significantly higher in several EMT-high tumors (**Figure 6B**). In addition, EMT-high tumors also showed increased activated stromal and hypoxia signatures. In Pancreatic Ductal Adenocarcinoma (PDAC), activated stroma signature has previously been associated with the worst outcome and macrophages (40). Furthermore, hypoxia can increase the expression of EMT-promoting transcription factors and is known to activate several pathways, including TGFβ, nuclear factor (NF)κB, and Notch signaling pathways, that promote EMT (41).

In SKCM, the expression of almost all cytokines and checkpoint molecules did not show a significant difference between EMT-high and EMT-low tumors. Furthermore, infiltration of macrophages was only moderately higher in “mesenchymal” SKCM. Even in EMT-high SKCM tumors, immune inhibitory cytokine expression was not significantly different from EMT-low counterparts. These differences of SKCM from other tumor types suggest that TIME of EMT-high SKCM tumors are not as inhibitory as in some other tumor types, such as LUAD and COADREAD, and may account for a high success rate of immunotherapy in SKCM (42).

Clustering analysis of different cancer types by TIME markers showed coclustering of most cancer types by EMT group. Furthermore, cancers could be classified into two groups based upon the differences in the median values of each TIME marker between EMT-high and -low groups (**Figure 8**). Previous studies analyzing TCGA data of various platforms (gene expression, methylation, reverse phase protein arrays, etc.) have shown coclustering of tumors with the tissue of origin. For example, coclustering was observed for gastrointestinal tumors including (COADREAD, STAD, and ESCA), kidney cancers (KIRP and KIRC), and squamous histology cancers (LUSC, HNSC, CESC, ESCA, and BLCA) (43–45). Our PCA did not show similar clustering; for example, lung cancers (LUSC and LUAD), head and neck cancers (HNSCC), and esophageal cancers (ESCA) were not clustering together in our analysis. Gastrointestinal tumors (COADREAD and STAD) were also not clustered together in our PCA (**Figure 8**). However, we did observe the coclustering of gynecological cancers (UESC and CESC).

These observations suggest that the changes of the TIME composition, consisting of change in proportions, phenotype, and function of infiltrating immune cells along with the presence of suppressive (high TGFβ and IL10) or exhausted immune cells (TIM3, LAG3, PD-1 expressing CD4+ and CD8+ T cells), might be required or facilitate the process of EMT. Indeed, an altered innate and adaptive immune response is known to play a pivotal role in enhancing tumor growth *via* selection of aggressive clones, induction of immunosuppression, and stimulation of cancer cell proliferation and metastasis (46). Overall, the crosstalk between enriched TAMs along with significant presence of naive and memory CD4+ T cells, cytotoxic CD8+ T cells, B cells, and Tregs may induce series of biochemical and molecular changes leading to generation of immune inhibitory components and creating permissive state of EMT.

In conclusion, our pan-cancer EMT analysis of 22 cancer types in TCGA dataset shows that the distinctive features of the EMT-high (mesenchymal) tumors are: (i) the enrichment of TAMs, (ii) overexpression of immune checkpoint molecules, and (iii) overexpression of immune inhibitory cytokines *TGFB1* and *IL10.* The role of TIME in anticancer immunity and immune checkpoint blockade failure is well recognized (47, 48). We have comprehensively analyzed the TIME of multiple cancer types in context to EMT and showed that TIME of the three EMT groups differs significantly. These findings will be helpful for future studies investigating the TIME and targeting TIME regulators for anticancer immunotherapy.

## Data Availability Statement

The original contributions presented in the study are included in the article/**Supplementary Material**. Further inquiries can be directed to the corresponding author.

## Ethics Statement

Ethical review and approval were not required for the study on human participants in accordance with the local legislation and institutional requirements. The patients/participants provided their written informed consent to participate in this study.

## Author Contributions

AK conceptualized the study, prepared figures and tables, and wrote the article. JT collected the data, carried out the analysis, and prepared the figures and tables. SNe participated in drafting and editing the article and assisted in the preparation of figures and tables. MK, SNi, and AS participated in figure preparation and drafting and editing the article. All authors contributed to the article and approved the submitted version.

## Funding

AK acknowledges the financial support from Department of Biotechnology, Government of India, in the form of Ramalingaswami Fellowship. The funding agency played no role in the study design, analysis, and interpretation of the results.

## Conflict of Interest

The authors declare that the research was conducted in the absence of any commercial or financial relationships that could be construed as a potential conflict of interest.

## Publisher’s Note

All claims expressed in this article are solely those of the authors and do not necessarily represent those of their affiliated organizations, or those of the publisher, the editors and the reviewers. Any product that may be evaluated in this article, or claim that may be made by its manufacturer, is not guaranteed or endorsed by the publisher.

## References

[1] Marcucci F Stassi G De Maria R. Epithelial-Mesenchymal Transition: A New Target in Anticancer Drug Discovery. Nat Rev Drug Discov (2016) 15:311–25. doi: 10.1038/nrd.2015.13 26822829

[2] Mittal V. Epithelial Mesenchymal Transition in Tumor Metastasis. Annu Rev Pathol Mech Dis (2018) 13:395–412. doi: 10.1146/annurev-pathol-020117-043854 29414248

[3] Thiery JP Acloque H Huang RYJ Nieto MA. Epithelial-Mesenchymal Transitions in Development and Disease. Cell (2009) 139(5):871–90. doi: 10.1016/j.cell.2009.11.007 19945376

[4] Roche J. The Epithelial-to-Mesenchymal Transition in Cancer. Cancers (Basel) (2018) 10(2):52. doi: 10.3390/cancers10020052 PMC5836084 29462906

[5] Jolly MK Boareto M Huang B Jia D Lu M Onuchic JN. Implications of the Hybrid Epithelial/Mesenchymal Phenotype in Metastasis. Front Oncol (2015) 5:155. doi: 10.3389/fonc.2015.00155 26258068 PMC4507461

[6] Sahoo S Nayak SP Hari K Purkait P Mandal S Kishore A. Immunosuppressive Traits of the Hybrid Epithelial/Mesenchymal Phenotype. bioRxiv (2021) 2021.06.21.449285. doi: 10.1101/2021.06.21.449285 PMC8714906 34975907

[7] Pastushenko I Mauri F Song Y de Cock F Meeusen B Swedlund B. Fat1 Deletion Promotes Hybrid EMT State, Tumour Stemness and Metastasis. Nature (2021) 589(7842):448–55. doi: 10.1038/s41586-020-03046-1 PMC7612440 33328637

[8] Terry S Savagner P Ortiz-Cuaran S Mahjoubi L Saintigny P Thiery J. New Insights Into the Role of EMT in Tumor Immune Escape. Mol Oncol (2017) 11(7):824. doi: 10.1002/1878-0261.12093 28614624 PMC5496499

[9] Hugo W Zaretsky JM Sun L Song C Moreno BH Hu-Lieskovan S. Genomic and Transcriptomic Features of Response to Anti-PD-1 Therapy in Metastatic Melanoma. Cell (2016) 165(1):35–44. doi: 10.1016/j.cell.2016.02.065 26997480 PMC4808437

[10] Chae YK Chang S Ko T Anker J Agte S Iams W. Epithelial-Mesenchymal Transition (EMT) Signature Is Inversely Associated With T-Cell Infiltration in Non-Small Cell Lung Cancer (NSCLC). Sci Rep (2018) 8(1):1–8. doi: 10.1038/s41598-018-21061-1 29440769 PMC5811447

[11] Aran D Hu Z Butte AJ. Xcell: Digitally Portraying the Tissue Cellular Heterogeneity Landscape. Genome Biol (2017) 18(1):1–14. doi: 10.1186/s13059-017-1349-1 29141660 PMC5688663

[12] Soundararajan R Fradette JJ Konen JM Moulder S Zhang X Gibbons DL. Targeting the Interplay Between Epithelial-to-Mesenchymal-Transition and the Immune System for Effective Immunotherapy. Cancers (Basel) (2019) 11(5):1–17. doi: 10.3390/cancers11050714 PMC6562947 31137625

[13] Lin Y Xu J Lan H. Tumor-Associated Macrophages in Tumor Metastasis: Biological Roles and Clinical Therapeutic Applications. J Hematol Oncol (2019) 12(1):1–16. doi: 10.1186/s13045-019-0760-3 31300030 PMC6626377

[14] Ravi J Elbaz M Wani NA Nasser MW Ganju RK. Cannabinoid Receptor-2 Agonist Inhibits Macrophage Induced EMT in Non-Small Cell Lung Cancer by Downregulation of EGFR Pathway. Mol Carcinog (2016) 55(12):2063–76. doi: 10.1002/mc.22451 PMC7063844 26741322

[15] Zhang J Yao H Song G Liao X Xian Y Li W. Regulation of Epithelial-Mesenchymal Transition by Tumor-Associated Macrophages in Cancer. Am J Transl Res (2015) 7(10):1699. 26692918 PMC4656751

[16] Chaplin DD. Overview of the Immune Response. J Allergy Clin Immunol (2010) 125(2 Suppl 2):S3. doi: 10.1016/j.jaci.2009.12.980 20176265 PMC2923430

[17] Speiser DE Ho PC Verdeil G. Regulatory Circuits of T Cell Function in Cancer. Nat Rev Immunol (2016) 16(10):599–611. doi: 10.1038/nri.2016.80 27526640

[18] Thomas MJ MacAry PA Noble A Askenase PW Kemeny DM. T Cytotoxic 1 and T Cytotoxic 2 CD8 T Cells Both Inhibit IgE Responses. Int Arch Allergy Immunol (2001) 124(1–3):187–9. doi: 10.1159/000053706 11306964

[19] Hanson HL Donermeyer DL Ikeda H White JM Shankaran V Old LJ. Eradication of Established Tumors by CD8 T Cell Adoptive Immunotherapy Competent Immune System to Tumor Tissue Can Result in the Generation of Specific Anti-Tumor Effectors (Dra-Noff Et al. Immunity (2000) 13:265–76. doi: 10.1016/S1074-7613(00)00026-1 10981969

[20] Kalams SA Walker BD. The Critical Need for CD4 Help in Maintaining Effective Cytotoxic T Lymphocyte Responses. J Exp Med (1998) 188(12):2199. doi: 10.1084/jem.188.12.2199 9858506 PMC2212425

[21] Romagnani S. Th1/Th2 cells. Inflamm Bowel Dis (1999) 5(4):285–94. doi: 10.1097/00054725-199911000-00009 10.1097/00054725-199911000-00009 10579123

[22] Ward-Hartstonge KA Kemp RA. Regulatory T-Cell Heterogeneity and the Cancer Immune Response. Clin Transl Immunol (2017) 6(9):e154. doi: 10.1038/cti.2017.43 PMC5628269 28983402

[23] Corthay A. How do Regulatory T Cells Work? Scand J Immunol (2009) 70(4):326. doi: 10.1111/j.1365-3083.2009.02308.x 19751267 PMC2784904

[24] Wartenberg M Zlobec I Perren A Koelzer VH Gloor B Lugli A. Accumulation of FOXP3+T-Cells in the Tumor Microenvironment Is Associated With an Epithelial-Mesenchymal-Transition-Type Tumor Budding Phenotype and Is an Independent Prognostic Factor in Surgically Resected Pancreatic Ductal Adenocarcinoma. Oncotarget (2015) 6(6):4190. doi: 10.18632/oncotarget.2775 25669968 PMC4414182

[25] Yang S Liu Y Li MY Ng CSH Yang S Wang S. FOXP3 Promotes Tumor Growth and Metastasis by Activating Wnt/β-Catenin Signaling Pathway and EMT in Non-Small Cell Lung Cancer. Mol Cancer (2017) 16(1):1–12. doi: 10.1186/s12943-017-0700-1 28716029 PMC5514503

[26] Landolt L Eikrem Ø Strauss P Scherer A Lovett DH Beisland C. Clear Cell Renal Cell Carcinoma Is Linked to Epithelial-To-Mesenchymal Transition and to Fibrosis. Physiol Rep (2017) 5(11):13305. doi: 10.14814/phy2.13305 PMC5471444 28596300

[27] Xu H Xu WH Ren F Wang J Wang HK Cao DL. Prognostic Value of Epithelial-Mesenchymal Transition Markers in Clear Cell Renal Cell Carcinoma. Aging (Albany NY) (2020) 12(1):866. doi: 10.18632/aging.102660 31915310 PMC6977664

[28] Tao C Huang K Shi J Hu Q Li K Zhu X. Genomics and Prognosis Analysis of Epithelial-Mesenchymal Transition in Glioma. Front Oncol (2020) 10:183. doi: 10.3389/fonc.2020.00183 32154177 PMC7047417

[29] Lou Y Diao L Cuentas ERP Denning WL Chen L Fan YH. Epithelial-Mesenchymal Transition Is Associated With a Distinct Tumor Microenvironment Including Elevation of Inflammatory Signals and Multiple Immune Checkpoints in Lung Adenocarcinoma. Clin Cancer Res (2016) 22(14):3630–42. doi: 10.1158/1078-0432.CCR-15-1434 PMC4947453 26851185

[30] Chaudhry A Samstein RM Treuting P Liang Y Pils MC Heinrich JM. Interleukin-10 Signaling in Regulatory T Cells Is Required for Suppression of Th17 Cell-Mediated Inflammation. Immunity (2011) 34(4):566–78. doi: 10.1016/j.immuni.2011.03.018 PMC3088485 21511185

[31] Thepmalee C Panya A Junking M Chieochansin T Yenchitsomanus Pt. Inhibition of IL-10 and TGF-β Receptors on Dendritic Cells Enhances Activation of Effector T-Cells to Kill Cholangiocarcinoma Cells. Hum Vaccines Immunother (2018) 14(6):1423–31. doi: 10.1080/21645515.2018.1431598 PMC6037468 29420117

[32] Wallet MA Sen P Tisch R. Immunoregulation of Dendritic Cells. Clin Med Res (2005) 3(3):166–75. doi: 10.3121/cmr.3.3.166 PMC1237158 16160071

[33] Xu J Lamouille S Derynck R. TGF-B-Induced Epithelial to Mesenchymal Transition. Cell Res (2009) 19(2):156–72. doi: 10.1038/cr.2009.5 PMC4720263 19153598

[34] Hao Y Baker D Dijke PT. TGF-β-Mediated Epithelial-Mesenchymal Transition and Cancer Metastasis. Int J Mol Sci (2019) 20(11):1–34. doi: 10.3390/ijms20112767 PMC6600375 31195692

[35] Kim BN Ahn DH Kang N Yeo CD Kim YK Lee KY. TGF-β Induced EMT and Stemness Characteristics Are Associated With Epigenetic Regulation in Lung Cancer. Sci Rep (2020) 10(1):10597. doi: 10.1038/s41598-020-67325-7 32606331 PMC7326979

[36] Komai T Inoue M Okamura T Morita K Iwasaki Y Sumitomo S. Transforming Growth Factor-β and Interleukin-10 Synergistically Regulate Humoral Immunity *via* Modulating Metabolic Signals. Front Immunol (2018) 9:1364. doi: 10.3389/fimmu.2018.01364 29963056 PMC6010538

[37] Fedele V Melisi D. Permissive State of EMT: The Role of Immune Cell Compartment. Front Oncol (2020) 10:587. doi: 10.3389/fonc.2020.00587 32391271 PMC7189417

[38] Ricciardi M Zanotto M Malpeli G Bassi G Perbellini O Chilosi M. Epithelial-To-Mesenchymal Transition (EMT) Induced by Inflammatory Priming Elicits Mesenchymal Stromal Cell-Like Immune-Modulatory Properties in Cancer Cells. Br J Cancer (2015) 112(6):1067–75. doi: 10.1038/bjc.2015.29 PMC4366889 25668006

[39] Romeo E Caserta CA Rumio C Marcucci F. The Vicious Cross-Talk Between Tumor Cells With an EMT Phenotype and Cells of the Immune System. Cells (2019) 8(5):460. doi: 10.3390/cells8050460 PMC6562673 31096701

[40] Moffitt RA Marayati R Flate EL Volmar KE Loeza SGH Hoadley KA. Virtual Microdissection Identifies Distinct Tumor- and Stroma-Specific Subtypes of Pancreatic Ductal Adenocarcinoma. Nat Genet (2015) 47(10):1168–78. doi: 10.1038/ng.3398 PMC4912058 26343385

[41] Tam SY Wu VWC Law HKW. Hypoxia-Induced Epithelial-Mesenchymal Transition in Cancers: HIF-1α and Beyond. Front Oncol (2020) 10:486. doi: 10.3389/fonc.2020.00486 32322559 PMC7156534

[42] Hamid O Robert C Daud A Hodi FS Hwu WJ Kefford R. Five-Year Survival Outcomes for Patients With Advanced Melanoma Treated With Pembrolizumab in KEYNOTE-001. Ann Oncol (2019) 30(4):582–8. doi: 10.1093/annonc/mdz011 PMC6503622 30715153

[43] Hoadley KA Yau C Hinoue T Wolf DM Lazar AJ Drill E. Cell-Of-Origin Patterns Dominate the Molecular Classification of 10,000 Tumors From 33 Types of Cancer. Cell (2018) 173(2):291–304.e6. doi: 10.1016/j.cell.2018.03.022 29625048 PMC5957518

[44] Chakraborty P Chen EL McMullen I Armstrong AJ Kumar Jolly M Somarelli JA. Analysis of Immune Subtypes Across the Epithelial-Mesenchymal Plasticity Spectrum. Comput Struct Biotechnol J (2021) 19:3842–51. doi: 10.1016/j.csbj.2021.06.023 PMC8283019 34306571

[45] Thorsson V Gibbs DL Brown SD Wolf D Bortone DS Ou Yang TH. The Immune Landscape of Cancer. Immunity (2018) 48(4):812–30. doi: 10.1016/j.immuni.2018.03.023 PMC5982584 29628290

[46] Palucka AK Coussens LM. The Basis of OncoImmunology. Cell (2016) 164(6):1233. doi: 10.1016/j.cell.2016.01.049 26967289 PMC4788788

[47] Binnewies M Roberts EW Kersten K Chan V Fearon DF Merad M. Understanding the Tumor Immune Microenvironment (TIME) for Effective Therapy. Nat Med (2018) 245 24(5):541–50. doi: 10.1038/s41591-018-0014-x PMC5998822 29686425

[48] Tang T Huang X Zhang G Hong Z Bai X Liang T. Advantages of Targeting the Tumor Immune Microenvironment Over Blocking Immune Checkpoint in Cancer Immunotherapy. Signal Transduct Target Ther (2021) 6(1):1–13. doi: 10.1038/s41392-020-00449-4 33608497 PMC7896069

