# Supplementary material for: Pan-Cancer Analysis Shows Enrichment of Macrophages, Overexpression of Checkpoint Molecules, Inhibitory Cytokines, and Immune Exhaustion Signatures in EMT-High Tumors
Source: Front Oncol. 2022 Jan 12;11:793881. doi: 10.3389/fonc.2021.793881 (PMC8790577; doi:10.3389/fonc.2021.793881)
Supplement: Supplementary file 1 [file DataSheet_1.docx]

Supplementary Material

1. **Supplementary Figures and Tables**
   1. **Supplementary Figures**

**Legends to Supplementary Figures**

**Supplementary Figure 1 |** Pan-cancer correlation analysis of EMT Score with the expression of **(A)** *TIM3* and **(B)** *TGFB1* in all three EMT groups.

**Supplementary Figure 2 |** Violin plots comparing expression of various markers in EMT-high, EMT-intermediate and EMT-low tumors across all cancer types. Violin plots of **(A)** CD4+ Naive T-cells, **(B)** *PD-1*, **(C)** *CTLA4*, **(D)** *SIGLEC5*, **(E)** *TGFB1*, **(F)** *IL10* and **(G)** *NOX2* are represented.

**Supplementary Figure 3 |** Pan-cancer correlation analysis of EMT Score with **(A)** 13-Genes inflammatory signature and **(B)** 9-Genes Exhausted CD8+ T-cells signature in all three EMT groups.

**Supplementary Figure 4 |** Silhouette width and K-means clustering of EMT-high and EMT-low patients of all cancer types. **(A)** Silhouette width analysis shows k =2 is optimum to cluster these cancer types. **(B)** K-means clustering with k=2 shows two separate clusters.

**Supplementary Figure 5 |** Survival of EMT-high and EMT-low patients across all cancer types. **(A)** Kaplan-Meier plots for Overall Survival (OS). **(B)** Kaplan-Meier plots for Progression Free Survival (PFS). The significant p-values are shown in red color and bold font.

**Supplementary Figure 6 |** Survival of EMT-high, EMT-intermediate and EMT-low patients of all cancer types. **(A)** Kaplan-Meier plots for OS. **(B)** Kaplan-Meier plots for PFS. The significant p-values are shown in red color and bold font.

- 1. **Supplementary Tables**

**Legends to Supplementary Tables**

**Supplementary Table 1 |** Details of all solid the cancer types in TCGA dataset. Of the 31 solid cancer types in TCGA dataset, 22 were included in the current study. THCA was not included because gene expression of *CDH1* gene, which is required for EMT score calculation, was missing. Other cancer types were excluded because of insufficient number of samples. Total number of samples in each cancer type, number samples in each EMT category (high, intermediate and low), and body site affected is also given.

**Supplementary Table 2 |** List of various immune cells analysed and Immune System component they belong.

**Supplementary Table 3 |** List of Immune checkpoint genes analysed with their symbols in TCGA dataset and currently HGNC approved symbol and name. The grouping column shows whether the gene is immune inhibitory or stimulatory in nature.

**Supplementary Table 4 |** List of various cytokines analysed with their symbols in TCGA dataset and currently HGNC approved symbol and name.

**Supplementary Table 5 |** Publicly available gene signatures analysed with the genes included in the signature and the corresponding reference.

**Supplementary Table 6 |** List of median values of various genes, expression signatures and immune cells enrichment scores used for the PCA shown in Figure 7A and Figure 8. Note that differences in the medians of EMT-high and EMT-low for each cancer type was used for Figure 7A.

**Supplementary Table 7 |** List of all gene signatures, checkpoints, cytokines and immune cells, sorted in order of their contributions to Principle Component 1 (PC1).

**Supplementary Table 2 | List of various immune cells analysed and Immune System component they belong.**

| **S.No** | **Immune Cell Name** | **Component (Innate or Adaptive)** |
| --- | --- | --- |
| 1. | Macrophages | Both |
| 2. | Monocytes | Both |
| 3. | Neutrophils | Innate |
| 4. | Natural Killer Cells | Innate |
| 5. | B cells | Adaptive |
| 6. | Memory B cells | Adaptive |
| 7. | CD4+ T Cells | Adaptive |
| 8. | CD4+ Naive T cells | Adaptive |
| 9. | CD4+ Memory T cells | Adaptive |
| 10. | Central Memory CD4+ T cells | Adaptive |
| 11. | Effector Memory CD4+ T cells | Adaptive |
| 12. | CD8+ T cells | Adaptive |
| 13. | Naive CD8+ T cells | Adaptive |
| 14. | Central Memory CD8+ T cells | Adaptive |
| 15. | Effector Memory CD8+ T cells | Adaptive |
| 16. | Type 1 Helper T cells | Adaptive |
| 17. | Regulatory T cells | Adaptive |

**Supplementary Table 3 | List of Immune checkpoint genes analysed with their symbols in TCGA dataset and currently HGNC approved symbol and name. The grouping column shows whether the gene is immune inhibitory or stimulatory in nature.**

| **S.No** | **Gene Symbol**  **(in TCGA)** | **HGNC approved Gene Symbol** | **HGNC Approved Gene Name** | **Grouping** |
| --- | --- | --- | --- | --- |
| 1. | SLEB2 | PD1 | Programmed cell death 1 | Checkpoint Inhibitory |
| 2. | CD274 | PD-L1 | Programmed cell death 1 ligand 1 | Checkpoint Inhibitory |
| 3. | PDCD1LG2 | PD-L2 | Programmed cell death 1 ligand 2 | Checkpoint Inhibitory |
| 4. | IDDM12 | CTLA4 | Cytotoxic T-lymphocyte associated protein 4 | Checkpoint Inhibitory |
| 5. | LAG3 | LAG3 | Lymphocyte activating 3 | Checkpoint Inhibitory |
| 6. | LOC115656 | KIR3DL1 | Killer cell immunoglobulin like receptor, three Ig domains and long cytoplasmic tail 1 | Checkpoint Inhibitory |
| 7. | HAVCR2 | TIM3 | Hepatitis A virus cellular receptor 2 | Checkpoint Inhibitory |
| 8. | C10orf54 | VISTA | V-set immunoregulatory receptor | Checkpoint Inhibitory |
| 9. | CYBB | NOX2 | cytochrome b-245 beta chain | Checkpoint Inhibitory |
| 10. | SIGLEC5 | SIGLEC5 | Sialic acid binding Ig like lectin 5 | Checkpoint Inhibitory |
| 11. | LOC100421066 | SIGLEC7 | Sialic acid binding Ig like lectin 7 | Checkpoint Inhibitory |
| 12. | SIGLEC15 | SIGLEC15 | Sialic acid binding Ig like lectin 15 | Checkpoint Inhibitory |
| 13. | FASLG | FASLG | Fas ligand | Checkpoint Inhibitory |
| 14. | ICOS | ICOS | Inducible T cell costimulator | Checkpoint Stimulatory |
| 15. | TNFRSF18 | GITR | TNF receptor superfamily member 18 | Checkpoint Stimulatory |
| 16. | TNFRSF4 | TNFRSF4 | TNF receptor superfamily member 4 | Checkpoint Stimulatory |
| 17. | TNFRSF9 | TNFRSF9 | TNF receptor superfamily member 9 | Checkpoint Stimulatory |

**Supplementary Table 4 | List of various cytokines analysed with their symbols in TCGA dataset and currently HGNC approved symbol and name.**

| **S.No** | **Gene Symbol (in TCGA)** | **HGNC approved Gene Symbol** | **HGNC approved Gene Name** |
| --- | --- | --- | --- |
| 1. | LOC89955 | IFNA1 | Interferon alpha 1 |
| 2. | IFNB1 | IFNB1 | Interferon beta 1 |
| 3. | IFNG.1 | IFNG | Interferon gamma |
| 4. | TNF | TNFA | Tumor necrosis factor |
| 5. | DPD1 | TGFB1 | Transforming growth factor beta 1 |
| 6. | IL1A | IL1A | Interleukin 1 alpha |
| 7. | IL1B | IL1B | Interleukin 1 beta |
| 8. | IL2 | IL2 | Interleukin 2 |
| 9. | IL3 | IL3 | Interleukin 3 |
| 10. | IL4 | IL4 | Interleukin 4 |
| 11. | IL5 | IL5 | Interleukin 5 |
| 12. | IL6 | IL6 | Interleukin 6 |
| 13. | CXCL8 | CXCL8 | C-X-C motif chemokine ligand 8 |
| 14. | IL10 | IL10 | Interleukin 10 |
| 15. | IL12A | IL12A | Interleukin 12A |
| 16. | BHR1 | IL12B | Interleukin 12B |
| 17. | STAT6 | STAT6 | Signal transducer and activator of transcription 6 |

**Supplementary Table 5 | Publicly available gene signatures analysed with the genes included in the signature and the corresponding reference.**

| **Signature Name** | **Gene Signatures** | **Supplementary Reference** |
| --- | --- | --- |
| **6-gene IFNG** | *CXCL9, STAT1, IDO1, CXCL10, HLA-DRA, IFNG* | (1) |
| **Activated Stroma** | *SPARC, COL1A2, COL3A1, POSTN, COL5A2, COL1A1, THBS2, FN1, COL10A1, COL5A1, SFRP2, CDH11, CTHRC1, FNDC1, SULF1, FAP, LUM, COL11A1, ITGA11, MMP11, INHBA, VCAN, GREM1, COMP* | (2) |
| **Macrophages** | *FN1, MSR1, CD68, CCL7, PPBP, CXCL5* | (3) |
| **3-gene exhausted CD8** | *LAG3, CD244, EOMES* | (4) |
| **9-gene exhausted CD8** | *CXCL3, LAG3, CCL5, CD244, CSF3R, CXCL13, CYBB, KLRK1, MSR1* | (4) |
| **Gajewski 13-gene Inflammatory** | *CXCL9, CD8A, CXCL10, CCL2, CCL3, CCL4, GAZMK, HLA-DMA, HLA-DMB, HLA-DOB, ICOS, IRF1, HLA-DOA* | (5) |
| **Cytolytic activity** | *GZMA, PRF1* | (6) |
| **15 gene Hypoxia Signature** | *VEGF, PGAM1, ENO1, LDHA, TPI1, P4HA1, MRPS17, ADM, NDRG1, TUBB6, ALDOA, MIF, SLC2A1, CDKN3, ACOT7* | (7) |

**Supplementary Table 7 | List of all gene signatures, checkpoints, cytokines and immune cells, sorted in order of their contributions to Principle Component 1 (PC1).**

| **Factor** | **PC1** | **PC2** |
| --- | --- | --- |
| Gajewski 13-gene Inflammatory (gene signature) | 4.35725715 | 0.07978419 |
| 9-gene exhausted CD8 (gene signature) | 4.33615616 | 0.1236718 |
| TIM3 | 4.10259167 | 0.1279546 |
| SIGLEC7 | 4.09278235 | 0.01127084 |
| PD-L2 | 3.97575232 | 0.00363891 |
| PD-1 | 3.94690093 | 0.1423294 |
| CTLA4 | 3.87723272 | 0.00288545 |
| 3-gene exhausted CD8 (gene signature) | 3.8765766 | 0.04076279 |
| Cytolytic activity | 3.85485453 | 0.00010519 |
| IL10 | 3.84657633 | 0.19651507 |
| ICOS | 3.82095649 | 0.18872925 |
| Macrophages (gene signature) | 3.8035889 | 0.00412935 |
| NOX2 | 3.64945296 | 0.29578686 |
| Merk 6-gene IFNG (gene signature) | 3.58679082 | 0.1417565 |
| FASLG | 3.3253696 | 0.14682885 |
| Activated stroma (gene signature) | 3.28736326 | 0.39105591 |
| LAG3 | 3.27336931 | 0.34418921 |
| TNFRSF4 | 3.20096509 | 0.01515155 |
| SIGLEC5 | 3.08407384 | 0.0054442 |
| TGFB1 | 3.02566724 | 0.11528979 |
| TNFRSF9 | 2.71312991 | 0.00021771 |
| TNFA | 2.59082421 | 0.19317735 |
| GITR | 2.52787552 | 0.00150686 |
| VISTA | 2.11890836 | 0.03381066 |
| IFNG | 2.00926401 | 0.19957416 |
| IL6 | 2.00612748 | 0.04018432 |
| IL12B | 1.65796257 | 0.6263266 |
| CXCL8 | 1.13735087 | 0.01502994 |
| Macrophages | 1.10746698 | 0.00955479 |
| Monocytes | 1.00258838 | 1.77293487 |
| 15 gene Hypoxia Signature (gene signature) | 0.87208605 | 0.29238093 |
| IL1B | 0.82639492 | 0.67798895 |
| STAT6 | 0.7159217 | 0.01494141 |
| IL2 | 0.70257072 | 0.36796172 |
| PD-L1 | 0.58384679 | 0.15872325 |
| IL12A | 0.45606878 | 0.50123424 |
| SIGLEC15 | 0.40881473 | 0.9704519 |
| Memory B cells | 0.40090663 | 0.13954496 |
| KIR3DL1 | 0.33099519 | 2.6231341 |
| Central Memory CD8+ T cells | 0.25318731 | 3.84679438 |
| Naive CD8+ T cells | 0.24448397 | 4.31621073 |
| IFNA1 | 0.16243366 | 1.8112216 |
| Regulatory T cells | 0.13419211 | 2.53555119 |
| CD4+ Memory T cells | 0.12591073 | 11.341442 |
| Effector Memory CD4+ T cells | 0.12021821 | 0.59885478 |
| Natural Killer Cells | 0.11263211 | 0.66858561 |
| B cells | 0.10319904 | 13.4183238 |
| IL1A | 0.09702226 | 0.16086391 |
| CD4+ T Cells | 0.03549631 | 2.87676865 |
| Neutrophils | 0.03478343 | 1.3145419 |
| IFNB1 | 0.03254089 | 3.13739285 |
| Naive CD8+ T cells | 0.02171943 | 11.8038058 |
| CD8+ T cells | 0.00883022 | 5.18527532 |
| IL5 | 0.00813945 | 0.00040298 |
| Central Memory CD4+ T cells | 0.005089 | 1.82386892 |
| Type 2 Helper T cells | 0.00444284 | 8.72973013 |
| Type 1 Helper T cells | 0.00127082 | 5.65846777 |
| IL4 | 0.0010233 | 1.92304104 |
| IL3 | 2.84E-06 | 7.83289826 |

**SUPPLEMENTARY REFERENCES**

1. Ayers M, Lunceford J, Nebozhyn M, Murphy E, Loboda A, Kaufman DR, et al. IFN-γ–related mRNA profile predicts clinical response to PD-1 blockade. J Clin Invest [Internet]. 2017 Aug 1 [cited 2021 Sep 19];127(8):2930–40. Available from: https://doi.org/10.1172/JCI91190.

2. Moffitt RA, Marayati R, Flate EL, Volmar KE, Loeza SGH, Hoadley KA, et al. Virtual microdissection identifies distinct tumor- and stroma-specific subtypes of pancreatic ductal adenocarcinoma. Nat Genet 2015 4710 [Internet]. 2015 Sep 7 [cited 2021 Sep 19];47(10):1168–78. Available from: https://www.nature.com/articles/ng.3398

3. Bindea G, Mlecnik B, Tosolini M, Kirilovsky A, Waldner M, Obenauf AC, et al. Spatiotemporal Dynamics of Intratumoral Immune Cells Reveal the Immune Landscape in Human Cancer. Immunity. 2013 Oct 17;39(4):782–95.

4. Hsu C-L, Ou D-L, Bai L-Y, Chen C-W, Lin L, Huang S-F, et al. Exploring Markers of Exhausted CD8 T Cells to Predict Response to Immune Checkpoint Inhibitor Therapy for Hepatocellular Carcinoma. Liver Cancer [Internet]. 2021 Jul 1 [cited 2021 Sep 19];10(4):346–59. Available from: https://www.karger.com/Article/FullText/515305

5. Spranger S, Bao R, Gajewski TF. Melanoma-intrinsic β-catenin signalling prevents anti-tumour immunity. Nat 2015 5237559 [Internet]. 2015 May 11 [cited 2021 Sep 19];523(7559):231–5. Available from: https://www.nature.com/articles/nature14404

6. Rooney MS, Shukla SA, Wu CJ, Getz G, Hacohen N. Molecular and Genetic Properties of Tumors Associated with Local Immune Cytolytic Activity. Cell [Internet]. 2015 Jan 15 [cited 2021 Sep 19];160(1):48–61. Available from: http://www.cell.com/article/S0092867414016390/fulltext

7. Buffa FM, Harris AL, West CM, Miller CJ. Large meta-analysis of multiple cancers reveals a common, compact and highly prognostic hypoxia metagene. Br J Cancer 2010 1022 [Internet]. 2010 Jan 19 [cited 2021 Sep 19];102(2):428–35. Available from: https://www.nature.com/articles/6605450
