# Supplementary figures and images for: Pan-Cancer Analysis Shows Enrichment of Macrophages, Overexpression of Checkpoint Molecules, Inhibitory Cytokines, and Immune Exhaustion Signatures in EMT-High Tumors
Source: Front Oncol. 2022 Jan 12;11:793881. doi: 10.3389/fonc.2021.793881 (PMC8790577; doi:10.3389/fonc.2021.793881)

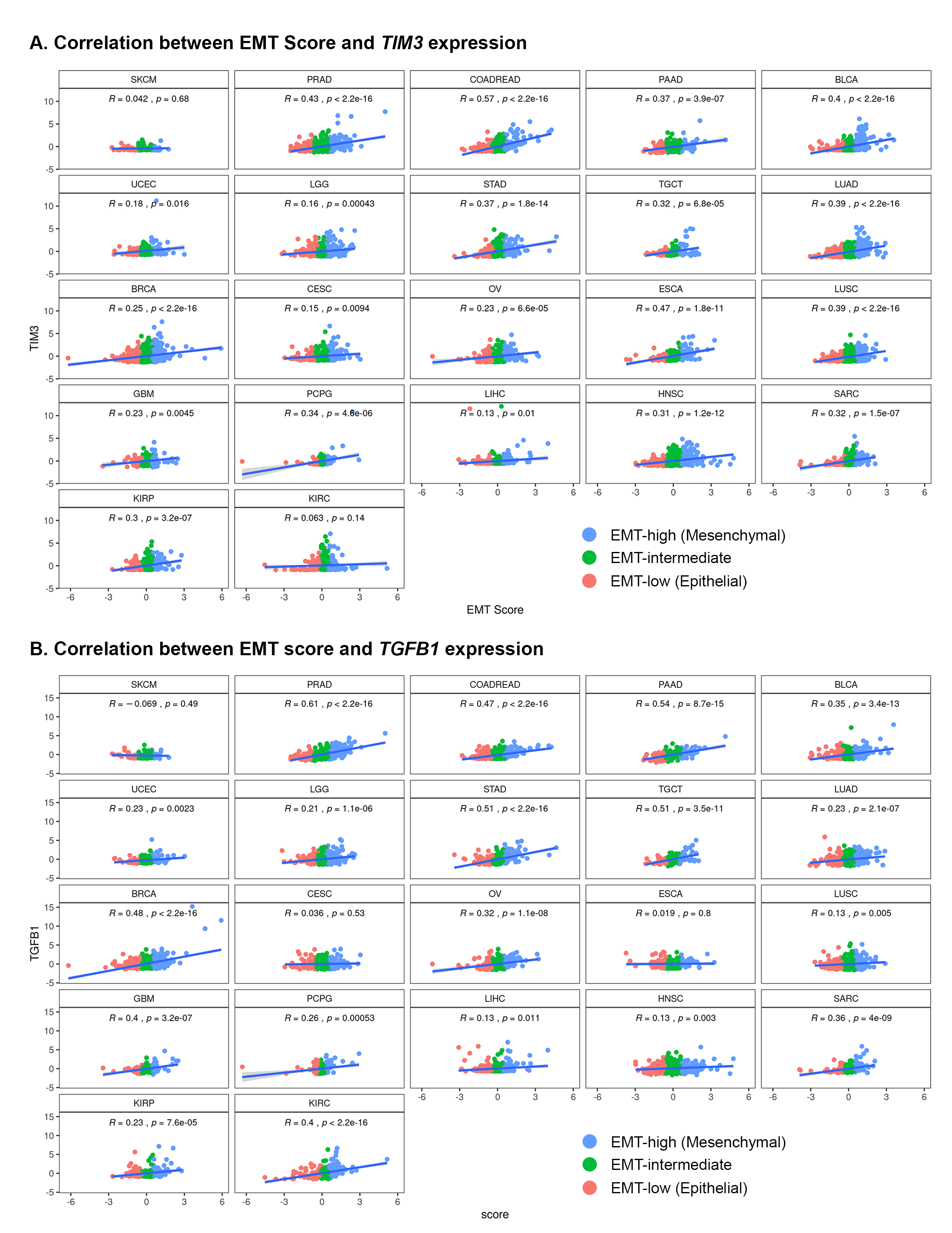

Supplement: Supplementary file 2 [file Image_1.jpeg]

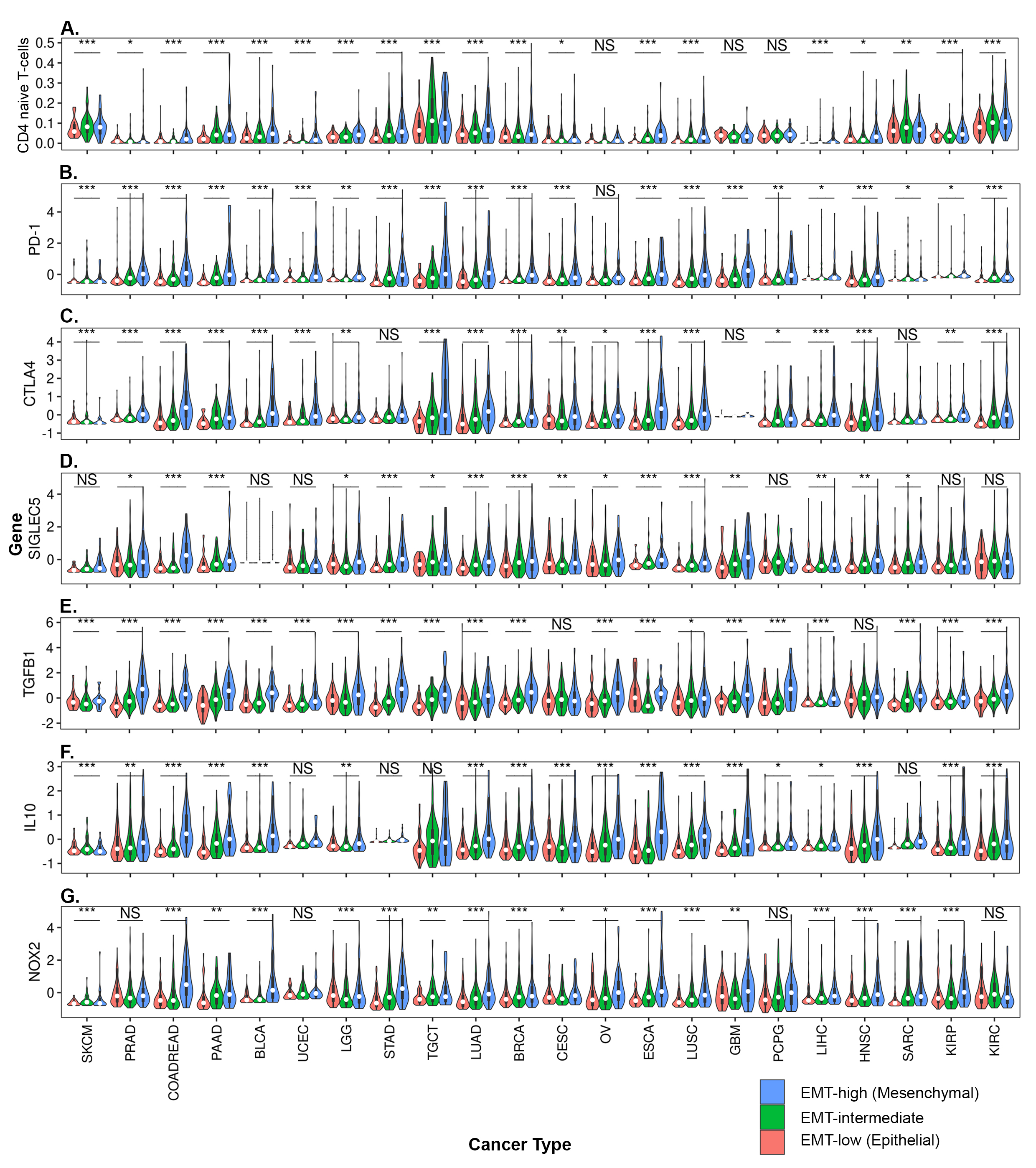

Supplement: Supplementary file 3 [file Image_2.jpeg]

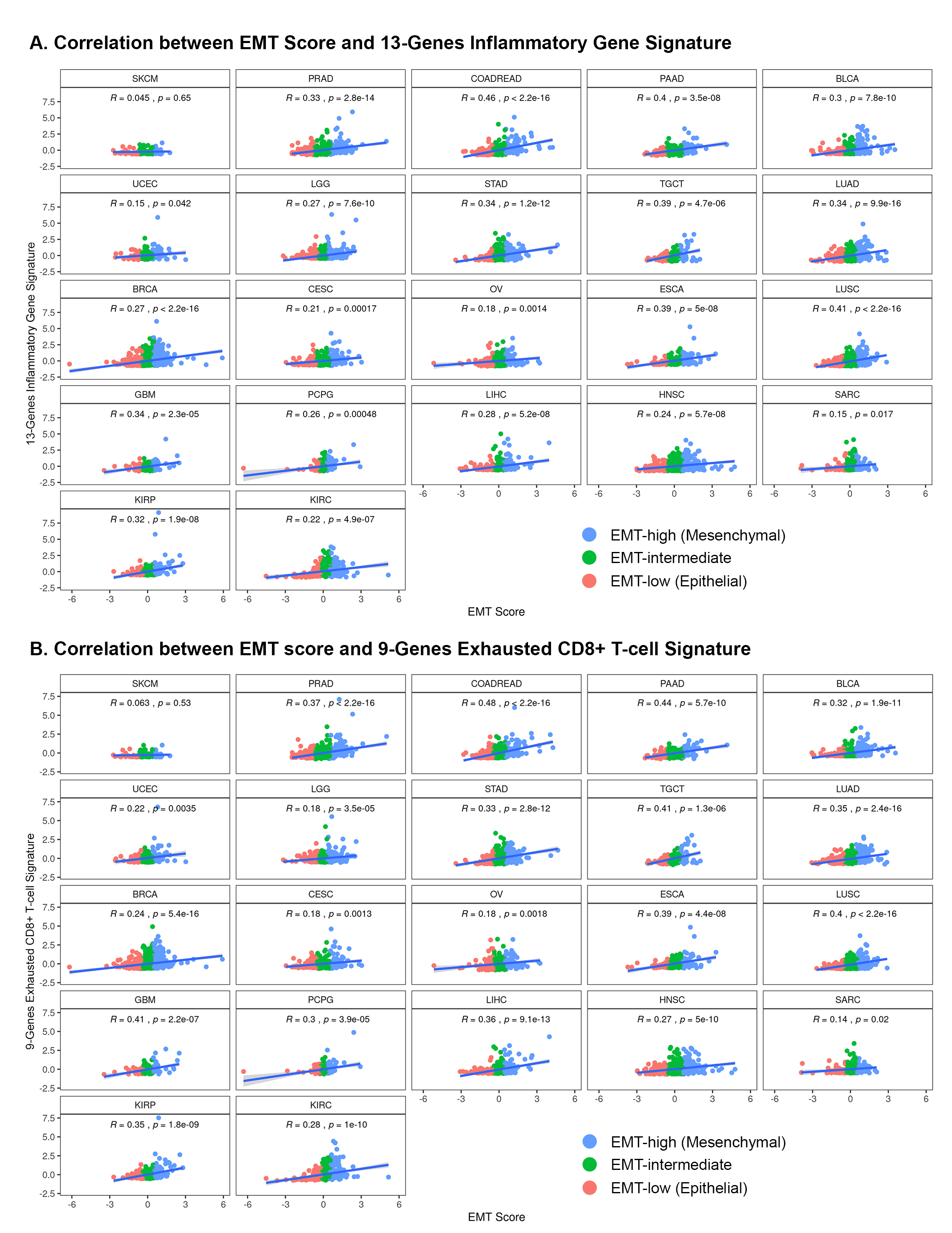

Supplement: Supplementary file 4 [file Image_3.jpeg]

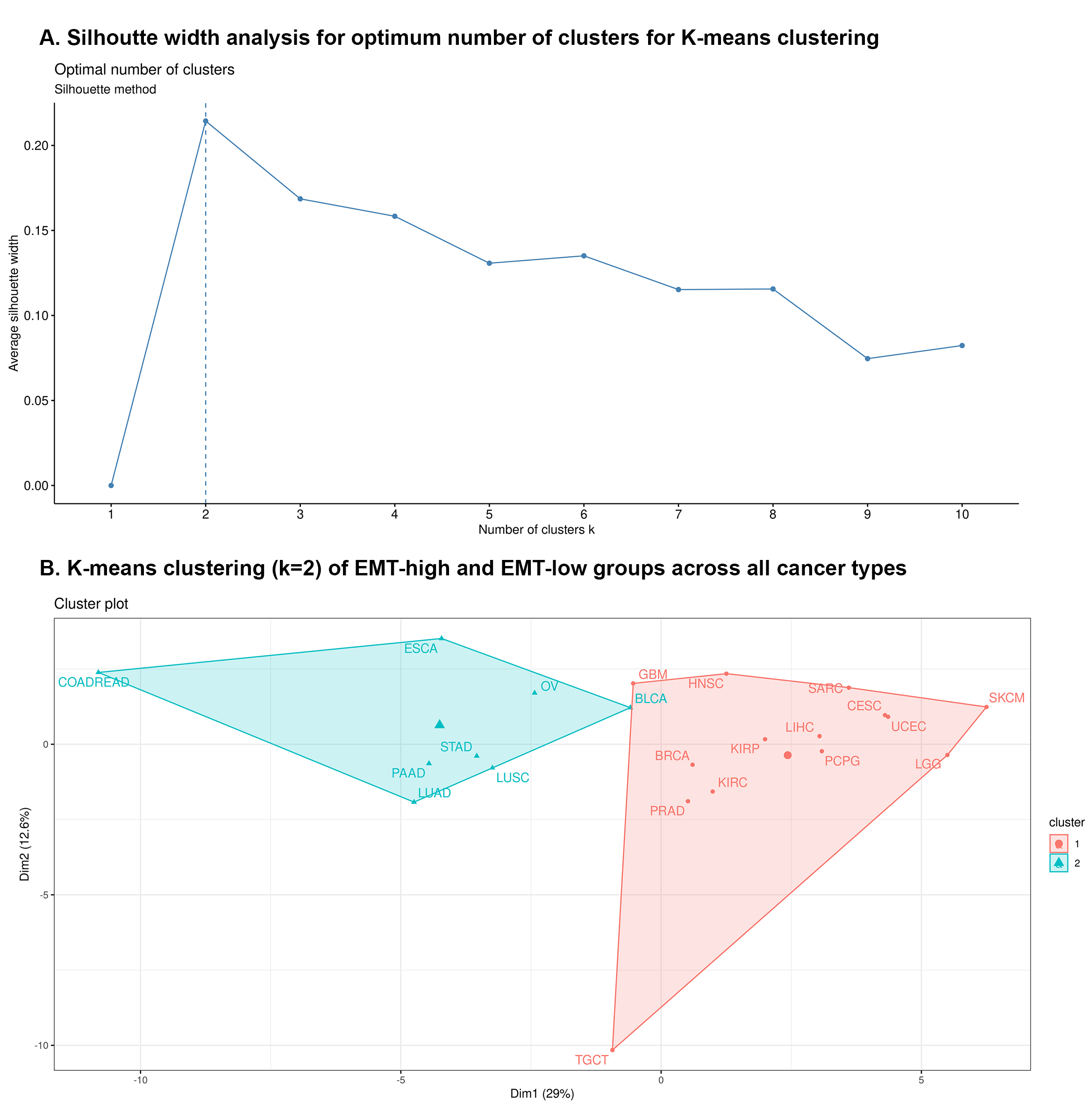

Supplement: Supplementary file 5 [file Image_4.jpeg]

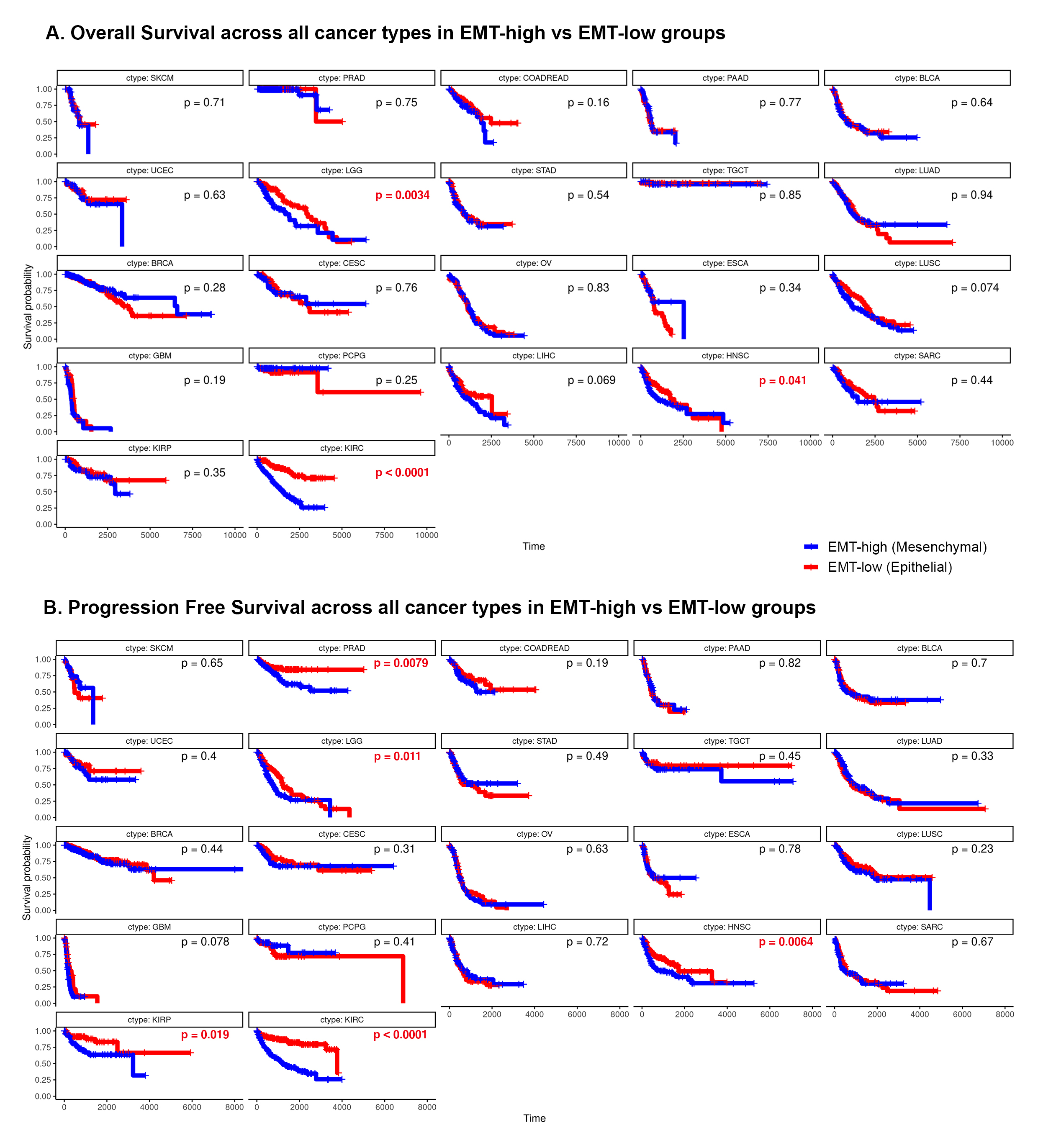

Supplement: Supplementary file 6 [file Image_5.jpeg]

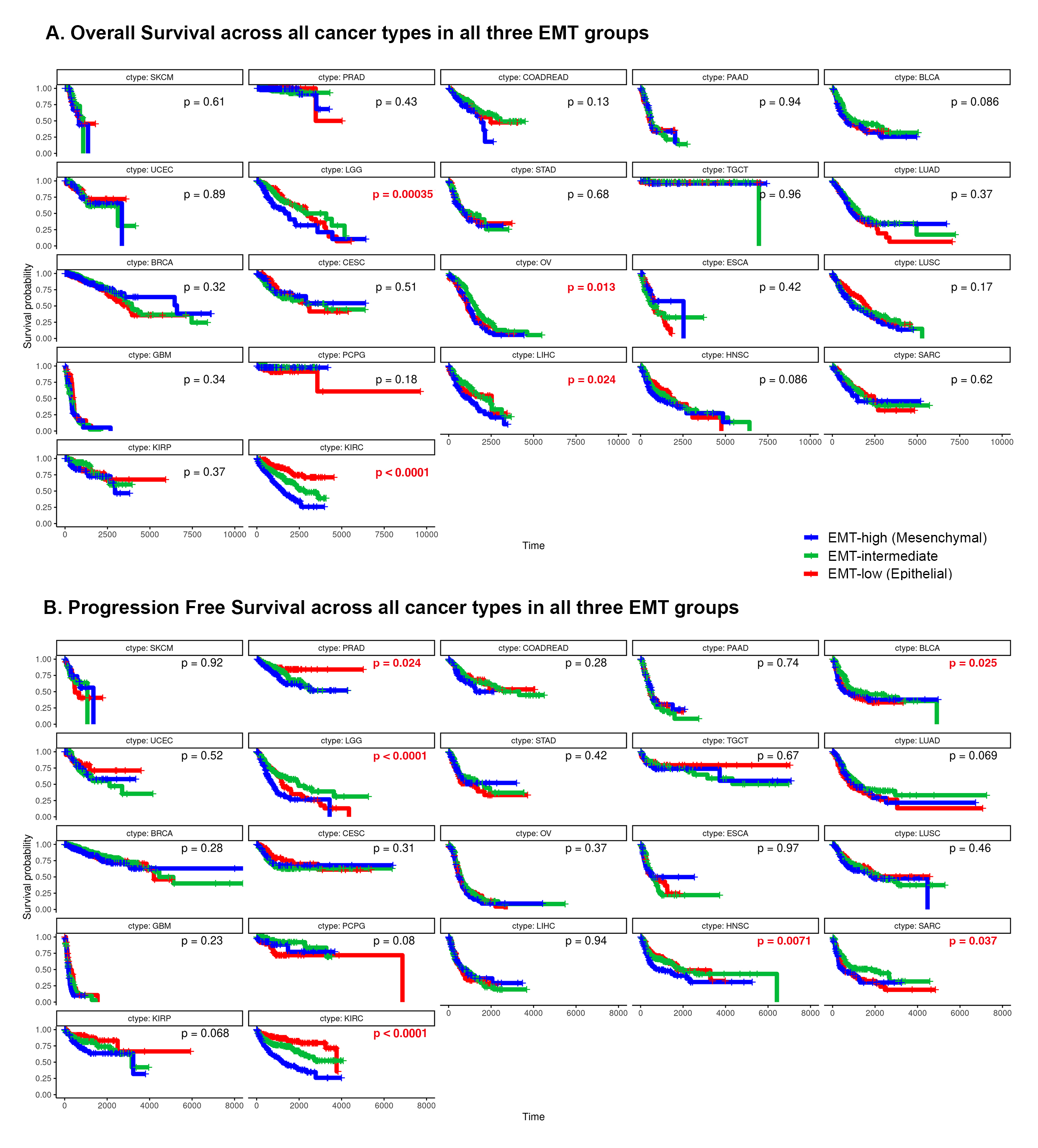

Supplement: Supplementary file 7 [file Image_6.jpeg]
